# Recognition of COVID-19 from CT Scans Using Two-Stage Deep-Learning-Based Approach: CNR-IEMN †

**DOI:** 10.3390/s21175878

**Published:** 2021-08-31

**Authors:** Fares Bougourzi, Riccardo Contino, Cosimo Distante, Abdelmalik Taleb-Ahmed

**Affiliations:** 1 Institute of Applied Sciences and Intelligent Systems, National Research Council of Italy, 73100 Lecce, Italy; fares.bougourzi@isasi.cnr.it (F.B.); riccardo.contino@isasi.cnr.it (R.C.); 2 Department of Innovation Engineering, University of Salento, 73100 Lecce, Italy; 3 Univ. Polytechnique Hauts-de-France, Univ. Lille, CNRS, Centrale Lille, UMR 8520, F-59313 Valenciennes, France; Abdelmalik.Taleb-Ahmed@uphf.fr

**Keywords:** deep learning, multi-tasks strategy, slice-level classification, COVID-19, CT scans

## Abstract

Since the appearance of the COVID-19 pandemic (at the end of 2019, Wuhan, China), the recognition of COVID-19 with medical imaging has become an active research topic for the machine learning and computer vision community. This paper is based on the results obtained from the 2021 COVID-19 SPGC challenge, which aims to classify volumetric CT scans into normal, COVID-19, or community-acquired pneumonia (Cap) classes. To this end, we proposed a deep-learning-based approach (CNR-IEMN) that consists of two main stages. In the first stage, we trained four deep learning architectures with a multi-tasks strategy for slice-level classification. In the second stage, we used the previously trained models with an XG-boost classifier to classify the whole CT scan into normal, COVID-19, or Cap classes. Our approach achieved a good result on the validation set, with an overall accuracy of 87.75% and 96.36%, 52.63%, and 95.83% sensitivities for COVID-19, Cap, and normal, respectively. On the other hand, our approach achieved fifth place on the three test datasets of SPGC in the COVID-19 challenge, where our approach achieved the best result for COVID-19 sensitivity. In addition, our approach achieved second place on two of the three testing sets.

## 1. Introduction

COVID-19 is a respiratory infection caused by the virus named SARS-CoV-2 belonging to the coronavirus family. The virus mainly affects the respiratory tract but can also cause symptoms affecting other organs [1]. In more than half of the cases, the infection is asymptomatic, and in about a third of the cases, it presents flu-like symptoms [2]. Since the appearance of COVID-19 in Wuhan, China, in 2019, much effort has been made to fend off the spreading of COVID-19 infection. To stop the spread of COVID-19, it is mandatory to recognize and then confine infected persons. Many recognition methods have proved their efficiency including RT-PCR, CT scans, and X-ray scans [3]. Despite the fact that the RT-PCR test is considered as the gold standard in diagnosing COVID-19, it has a considerable false-negative rate, especially in early stages of infection [4]. In contrast, using X-ray scan and CT scan methods can give efficient results in both time and accuracy [3]. In fact, using CT scans and X-ray scans requires an expert radiologist to identify the COVID-19 infection. Artificial intelligence (AI) systems can provide an alternative solution for automatic diagnosis of COVID-19 infections [3,5].

This paper is an extended version for our IEEE International Conference on Acoustics, Speech, and Signal Processing (ICASSP) paper [6] for the 2021 COVID-19 SPGC challenge [7]. The objective of this work is to give more details about each step of our proposed approach. Furthermore, we highlight all experiments that we have conducted in developing our approach. In addition, we discuss the results of each experiment to provide more understanding of our work. To have more insight into the trained CNN models, we used heat maps to check if the trained models were looking in the right places in the CT scan slices to classify them.

To evaluate the performance of our approach, we used the SPGC-COVID dataset [8], which contains volumetric chest CT scans of 171 patients positive for COVID-19, 60 with community-acquired pneumonia (CAP), and 76 normal cases. In addition to the train and val splits, there are three testing splits with a total of 90 CT scans, and each testing set has specific characteristics and challenges.

To classify the CT scans into COVID-19, CAP, and normal, we proposed an ensemble deep learning approach. Our CNR-IEMN approach consists of two stages. In the first stage, we fine-tuned deep learning models using a multi-tasks strategy for slice-level classification. In the second stage, the entire volume of the CT scan is classified using slice-level predictions and the XG-boost classifier. In summary, the main contributions of this paper are:

- We propose to segment the lung lobes of the slice, and then we stack the original slice channel with the segmented channels to obtain an RGB-like image.
- With the use of the multi-tasks strategy and data augmentation for COVID-19 and Cap slices, we used transfer learning to train four convolutional neural network (CNN) architectures (ResneXt-50, Densenet-161, Inception-v3, and Wide-Resnet-50) for slice-level classification.
- For patient-level CT scan classification, we propose to divide the CT scan slices into groups and then calculate the percentage of each class within each group using all trained CNN models for the slice-Level classification. In the end, we combined all grouping percentages from all trained CNN architectures and then fed them into an XG-boost classifier [9] to recognize the CT scan category.

This paper is organized in following way: Section 2 describes the related works. Section 3 contains the description of the materials and methods used. The experiments, results, and discussion are given in Section 4. Finally, we conclude our paper in Section 5.

## 2. Related Works

Since the appearance of COVID-19, CT scans have been widely used not only to recognize infected patients with COVID-19 but also to quantify the infection and monitor the evolution of the disease [5,10]. In the computer vision community, many approaches and frameworks have been proposed to help in recognizing and monitoring COVID-19 infection [5]. Since the deep learning approach has proved its efficiency in most computer vision tasks [11] (including medical imaging tasks [3,12]), most of the approaches that have been proposed for COVID-19 analysis from CT scans are CNN-based approaches. In general, the approaches that use CT scans for COVID-19 analysis can be classified into: segmentation [13,14,15] and recognition [16,17,18,19,20] approaches.

In [13], Q. Yan et al. proposed a feature variation block which adaptively adjusts the global properties of the features for segmenting COVID-19 infection. Their proposed feature variation block can enhance the capability of feature representation effectively. In addition, they fused features at different scales by proposing Progressive Atrous Spatial Pyramid Pooling to handle the sophisticated infection areas with diverse appearance and shapes. D. Müller et al. [14] proposed an automatic segmentation framework for COVID-19 infection segmentation from limited training data. In their approach, they investigated several preprocessing methods and exploiting extensive data augmentation. Moreover, they implemented a 3D U-Net architecture for both lungs and COVID-19-infected region segmentation.

In [16], H. Alshazly et al. investigated different deep learning architectures to classify CT scan images into COVID-19/non-COVID-19 using transfer learning and the LAMB optimizer on two databases. Furthermore, they provided visual explanations of the their trained models for detecting COVID-19-infected regions and compared them with expert radiologist annotations. N. Lassau et al. used 58 clinical and biological variables and chest CT scan data to predict the COVID-19 severity score [19]. They trained a CNN architecture using the CT scans to predict severity, and then they constructed the multimodal AI-severity score that includes five clinical and biological variables (age, sex, oxygenation, urea, and platelet) in addition to the deep learning model.

In addition to the two main tasks (COVID-19 segmentation and recognition), there are some works that proposed combining both tasks, such as [21,22]. In [21], C. Zheng et al. proposed a weakly supervised deep-learning-based framework that uses volumetric chest CT scans to detect COVID-19. First, the lung regions were segmented using a pre-trained UNet; then, the segmented 3D lung regions were fed into a 3D deep neural network to predict if the CT scan contained COVID-19 infection or not.

In the 2021 COVID-19 SPGC challenge [7], six conference papers were accepted, which corresponded to the approaches that achieved the best results [23,24,25,26,27]. TheSaviours [25] team proposed a two-stage convolutional neural network approach. The IITDelhi team [26] used a residual network at multiple scales to extract features from all the slices of the CT scan for each patient. Then, they used these features to train a patient-level classifier. The LLSCP team [24] proposed a multi-stage progressive learning strategy based on a 3D ResNet module. The UniSheff_EEE team [27] exploited transfer learning of a 3D Network (3D ResNet-50) to classify the volumetric CT scans. The Bingo team [27] proposed a novel ensemble learning framework with adaptive boosting and dataset clustering algorithms.

## 3. Materials and Methods

### 3.1. Database

The 2021 COVID-19 SPGC challenge [7] provided SPGC-COVID dataset [8] for the evaluation of the participants’ approaches. SPGC-COVID dataset [8] contains volumetric chest CT scans of patients positive for COVID-19 infection, community-acquired pneumonia (CAP), and normal patients. Each CT scan consists of all slices of the CT scan in the Digital Imaging and Communications in Medicine (DICOM) format, with a size of 512 × 512. The COVID-19 cases were collected from February 2020 to April 2020, whereas CAP cases and normal cases were collected from April 2018 to December 2019 and January 2019 to May 2020, respectively. Diagnosis of COVID-19 infection is based on positive real-time reverse transcription polymerase chain reaction (rRT-PCR) test results, clinical parameters, and CT scan manifestations identified by three experienced thoracic radiologists. It should be noted that each CT scan comes from a different patient, so the number of persons equals the number of CT scans.

In 2021 COVID-19 SPGC challenge [7], the dataset contains three splits: train, val, and test. The labels of the CT scans are known for the train and val Splits, while the test scan labels are unknown for all participants. The train and val sets were obtained from 307 volumetric chest CT scans (171 patients positive for COVID-19, 60 with community-acquired pneumonia (CAP), and 76 normal cases). A total of 30% of these 307 CT scans were selected randomly as validation set, and the remaining 70% were used as training set. All training and validation CT scans were obtained by SIEMENS, SOMATOM Scope scanner with the normal radiation dose and the slice thickness of 2 mm. Besides the patient-level labels, a subset of 55 COVID-19 and 25 CAP cases were analyzed by one radiologist to identify and label slices with evidence of infection.

In addition to the train and val splits, there were three testing sets:

1. Thirty CT scans of COVID-19, CAP, and normal cases from the same center where the training/validation sets were obtained.
2. Thirty low-dose CT scans (LDCT) of COVID-19 and normal cases obtained with slice thickness of 2 mm.
3. Thirty CT scans of COVID-19, CAP, and normal with a history of heart disease or operation in which an abnormal manifestation related to a non-infection disease is demonstrated in most (not all) of the cases. This set was obtained with various slice thickness and radiation doses.

### 3.2. Segmentation

**Figure 1:**
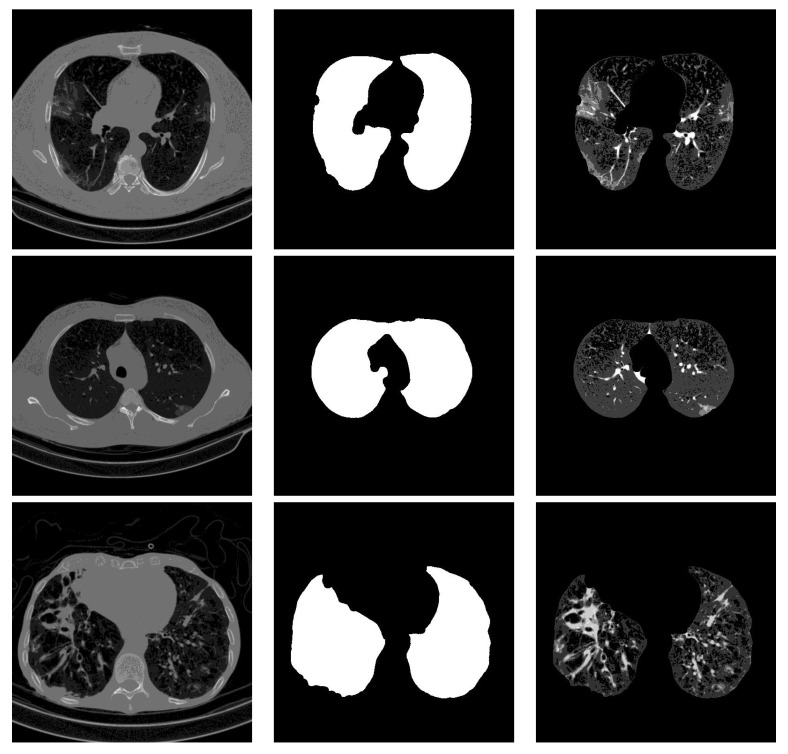
Lung segmentation examples: the first column shows the input CT scan slice, the second column shows the lungs mask result, and the last column shows the lung segmentation results. The corresponding classes for rows 1 to 3 are COVID-19, COVID-19, and Cap, respectively.

For lung segmentation, we used marker-based watershed segmentation [28], which is based on identifying two markers. The internal marker identifies the lung tissue, and the external marker identifies the outside of the region of interest. The internal marker is obtained by thresholding the image and removing all regions and leaving just the biggest one. The external marker is created by morphological dilation of the internal marker with two different iterations and subtracting the results. A watershed marker is created superimposing the two markers with different gray-scale values. To find the precise border of the lung, the marker-based watershed algorithm is applied on the black strip of the watershed marker and the Sobel gradient image of the original scan. In order not to miss lobes located next to the border regions, a black top-hat operation is performed to re-include those areas and areas surrounding the lung hila. Finally, the segmented lungs mask is obtained by holes closing. Figure 1 shows some examples of the lung segmentation for infected slices with COVID-19 and Cap diseases.

### 3.3. Our Proposed Approach

**Figure 2:**
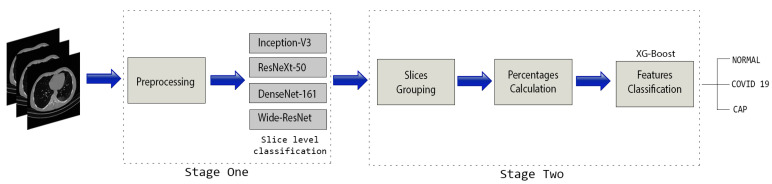
The overall structure of our proposed CNR-IEMN approach [6].

The overall structure of our approach is summarized in Figure 2. In summary, the proposed approach consists of two main stages. The main focus of the first stage is to classify the slices into one of the three classes (normal, COVID-19, or Cap). To this end, we used the training and validation CT scans that have the slice-level labels for both COVID-19 and Cap classes. Furthermore, we selected the first 30 normal CT scans and labeled all their slices as normal class. Since these CT scans are labeled as normal class, no slice within these CT scans contains COVID-19 or Cap infection. In total, we have 30, 55, and 24 CT scans for normal, COVID-19, and Cap, respectively. These CT scans were used to train and test deep learning architectures for slice-level classification. Among these CT scans, we have 9, 17, and 8 validation CT scans that have the slice level labeling. In the second stage, we predicted the slice label for the whole CT scan using trained CNN architectures. Since the number of slices of the CT scans varies from one CT scan to another, we propose dividing the slices into 20 equal groups. For each group, we calculate the slice prediction percentage of each class. By concatenating the percentages of all CT scan groups, we create a feature vector, which will be fed into an XG-boost classifier [9] to predict the class of the CT scan.

#### 3.3.1. Slice-Level Classification Stage

The aim of this stage is to fine-tune the CNN pretrained models to classify the CT scan slices into normal, COVID-19, or Cap class. The slice-level classification is the most important stage in our approach which can influence the whole CT scan prediction.

**Figure 3:**
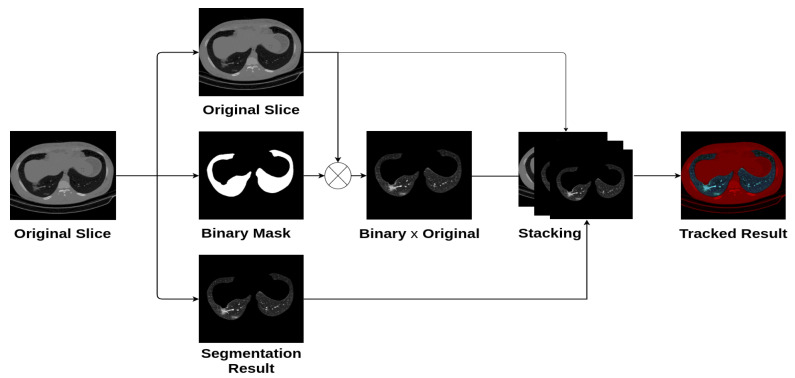
Preprocessing phase.

In the preprocessing phase, we read the slice images from the “.dcm” file format which gives one channel image (grayscale), where we did not use any contrast-enhancement technique. Since most CNN architectures were designed for color images, we propose stacking the gray image, the segmented lung lobes image, using the proposed method in [28] and the multiplication of the grayscale image with the binary lung lobes mask. Figure 3 shows an example of the original image, the segmentation result, and the result of stacking the three channels. In addition to having three channels as input to the CNN architectures, the segmentation guides the slices’ classification by concentrating on the lung lobes’ features and removing non-relevant ones.

To train the slice-based classification, we have 10,294, 2482, and 742 slices for normal, COVID-19, and Cap classes, respectively. From the number of slices of each class, we notice they are not balanced. To deal with this issue, we used data augmentation techniques for the COVID-19 and Cap slices. The data augmentation techniques used are:

- Color jitter with brightness = 0.2, contrast = 0.2, and applying probability = 0.2.
- Random horizontal flip with applying probability = 0.2.
- Random perspective with distortion scale = 0.5.
- Random rotation from −30 to 30 degrees.
- Random cropping.

For COVID-19 class, we generated 3 augmented images from each slice. On the other hand, we generated 10 augmented images for each Cap slice. In total, we obtained 9928 and 8162 for COVID-19 and Cap, respectively.

**Figure 4:**
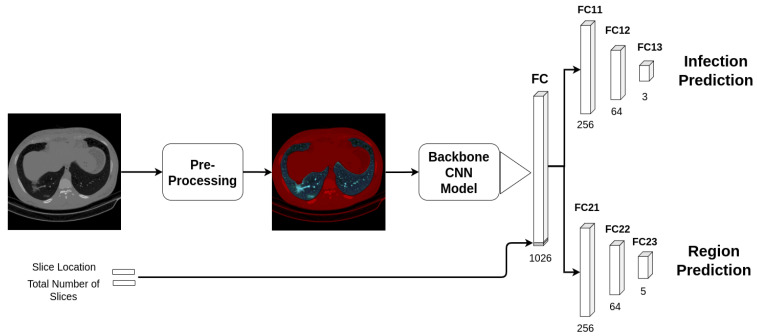
Multi-tasks slice-level training using CNN architectures.

**Figure 5:**
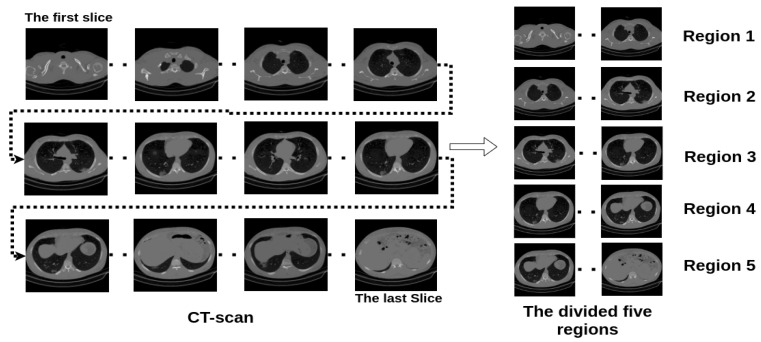
Example of dividing a CT scan into five regions.

Figure 4 shows an example of how we trained the four backbone CNN architectures, which are: ResneXt-50 [29], Densenet-161 [30], Inception-v3 [31], and Wide-Resnet-50 [32] (the backbone CNN architectures are the pre-trained models that were trained on ImageNet challenge database [33]). In our approach, we trained all of the CNN architectures using multi-tasks strategy. In more detail, we fine-tuned the CNN pretrained models, where just the FC layers were randomly initialized using uniform distribution initialization. The first task of the multi-tasks training is the classification of the input image into normal, COVID-19, and Cap classes. Since the size and the shape of the lungs change from the first, middle, and last slices of the CT scan, we divide each CT scan into five equalized regions as shown in Figure 5. The slices within the same region in the CT scan will have the same label. In addition to the main task (to classify the slices into normal, COVID-19, and Cap), the second task classifies the slice into one of the region classes. For the same purpose, we add the total number of slices of the CT scan and the slice location in the CT scan into the deep features (FC layer) as shown in Figure 4.

#### 3.3.2. Patient-Level Classification Stage

Since the CT scans have different number of slices, we propose dividing the CT scan into 20 groups where each group contains the same number of slices. After that, we applied our trained slice-level architectures to predict the labels of the slices of each group; then, we calculate the predicted slices percentage of each class within the group. This produces 20×3 = 60 features for each CT scan using one of the trained CNN architectures. For classifying the CT scans, we feed the combination of all trained CNN architecture features, by concatenating them alongside each other, into an XG-boost classifier [9]. In our experimental part, we trained the XG-boost with the training CT scan features; then, we evaluated the performance of our approach using the validation CT scan features.

## 4. Results and Discussion

### 4.1. Experimental Setup

For deep learning training and testing, we used the Pytorch [34] library with NVIDIA GPU Device GeForce TITAN RTX 24 GB. The batch size used consists of 64 images, and as a loss function, we used the focal loss function [35] with gamma equal to 1.5.

### 4.2. Slice-Level Classification

For slice-level classification, four powerful CNN architectures were used: ResneXt-50, Densenet-161, Inception-v3, and Wide-Resnet-50. Since slice-level classification is the most important stage in our approach, we investigated different scenarios. The investigated scenarios include data prepossessing, data augmentation, and training deep learning architectures with the multi-tasks strategy.

#### 4.2.1. Stacking Grayscale Image

Since the slice read from the ’.dcm’ file is a one-channel grayscale image and the input of the pretrained CNN models (on ImageNet) includes three channels (RGB images), we stacked the grayscale slice to obtain an RGB-like image. In this experiment, all CNN architectures were fully trained using transfer learning for 20 epochs with the Adam optimizer [36]. The initial learning rate is 0.0001, which decays by 0.1 after 10 epochs, followed by another decay of 0.1 after 15 epochs.

**Figure 6:**
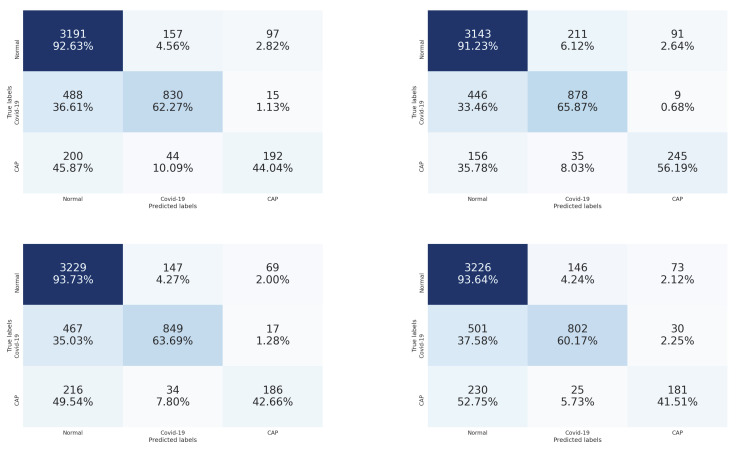
Confusion matrices of the stacked grayscale images on validation data using ResneXt-50, Inception-v3, DenseNet-161, and Ensemble-CNNs, respectively. The vertical axis is for the true classes, and the horizontal axis is for the predicted classes.

Table 1 summarizes the obtained results. From these results, we notice that the four CNN architectures achieved similar results with slightly better results using the Densenet-161 architecture. Figure 6 contains the confusion matrices of the trained CNN architectures (ResneXt-50, Densenet-161, Inception-v3, and Wide-Resnet-50) on the validation data. From these confusion matrices, we notice that the Densenet-161 architecture achieved the best performance in the recognition of COVID-19 and Cap slices with accuracies of 65.87% and 56.19%, respectively. On the other hand, for normal slice recognition, the Inception-v3 architecture achieved the best performance with an accuracy of 93.73%.

#### 4.2.2. Stacking Grayscale Image with the Segmented Result

In this experiment, we proposed segmenting the lung lobes and then stacking the segmentation result with the raw slice image to obtain an RGB-like image as illustrated in Figure 3. Similar to the stacking grayscale experiment, all CNN models were fine-tuned for 20 epochs with the Adam optimizer [36]. The initial learning rate is 0.0001, which decays by 0.1 after 10 epochs, followed by another decay of 0.1 after 15 epochs. Table 2 summarizes the obtained results of the four CNN architectures. From these results, we notice that the Densenet-161 architecture achieved a slightly better result than the other three CNN architectures. In general, the four CNN architectures achieved close results.

**Figure 7:**
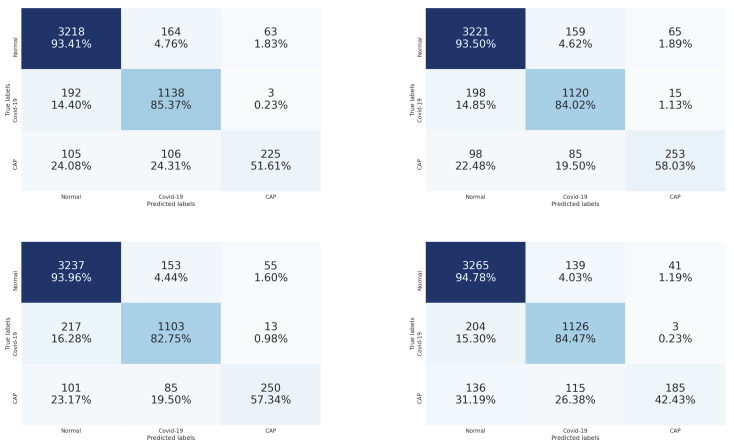
Confusion matrices of the stacked grayscale image and the segmented lung lobes on validation data using ResneXt-50, Densenet-161, Inception-v3, and Wide-Resnet-50, respectively. The vertical axis is for the true classes, and the horizontal axis is for the predicted classes.

Figure 7 contains the confusion matrices of the trained CNN architectures (ResneXt-50, Densenet-161, Inception-v3, and Wide-Resnet-50) on the validation data. From these confusion matrices, we notice that the Wide-Resnet-50 architecture achieved the best performance on the recognition of normal and COVID-19 slices with accuracies of 94.78% and 84.47%, respectively. On the other hand, for Cap slices recognition, the Densenet-161 architecture achieved the best performance with an accuracy of 58.03%.

By comparing the results of Table 1 and Table 2, we notice that stacking the segmentation of lung lobes considerably improved the results of all four CNN architectures. Similarly, when we compare the confusion matrices of Figure 6 and Figure 7, we notice that the best recognition rate of all three classes improved, especially for the COVID-19 rate, which improved by 18.6%. This proves the importance of stacking the segmented lung lobes with the grayscale image in our approach.

#### 4.2.3. Data Augmentation of the Stacked Gray and Segmented Images Scenario

In this experiment, we proposed augmenting the stacked gray and segmented lung lobes to have a balanced database as described in Section 3.3.1. Since the data are augmented in this scenario, we fine-tuned the CNN models for only 10 epochs with the Adam optimizer [36]. The initial learning rate is 0.0001, which decays by 0.1 after 3 epochs, followed by another decay of 0.1 after 6 epochs. Table 3 summarizes the obtained results of four CNN architectures using the augmented data. From these results, we notice that the Inception-v3 architecture achieved a slightly better result than the other three CNN architectures. In comparison with the results of Table 2, we notice that the performance of the four CNN architectures improved.

**Figure 8:**
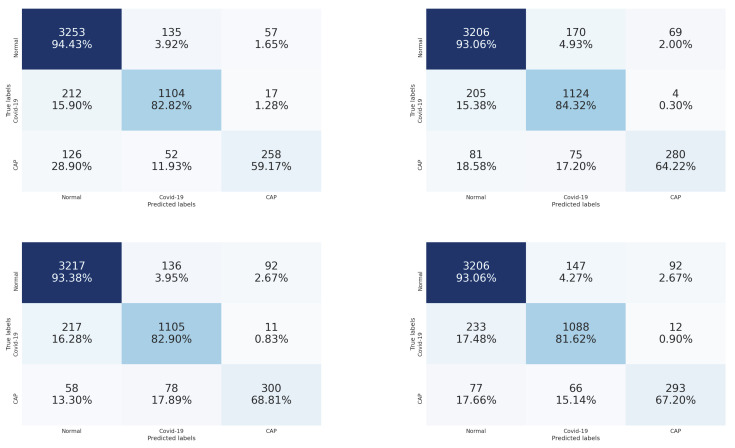
Confusion matrices of the augmented stacked grayscale image and the segmented lung lobes on validation data using ResneXt-50, Densenet-161, Inception-v3, and Wide-Resnet-50, respectively. The vertical axis is for the true classes, and the horizontal axis is for the predicted classes.

Figure 8 contains the confusion matrices of the trained CNN architectures (ResneXt-50, Densenet-161, Inception-v3, and Wide-Resnet-50) on the validation data. From these confusion matrices, we notice that the ResneXt-50, Inception-v3, and Densenet-161 architectures achieved the best performance on the recognition of normal (94.43%), COVID-19 (84.32%), and Cap slices (68.81%), respectively. In this scenario, we observe that the recognition rate of the Cap slices has improved by about 10% compared with the previous scenario. This proves the efficiency of using data augmentation techniques.

#### 4.2.4. Multi-Tasks Experiment

In this experiment, we trained the augmented data of the stacked gray and segmented scenario using the multi-tasks paradigm as explained in Section 3.3.1. The first task is to classify the input stacked image (gray and segmented) into normal, COVID-19, and Cap classes. Since the size and the shape of the lungs change from the first, middle, and last slices of the CT scan, we divided each CT scan into five equalized regions. The slices within the same region in the CT scan will have the same label. In addition to the main task (to classify the slices into normal, COVID-19, and Cap), the second task classifies the slice into one of the region classes. For the same purpose, we added the total number of slices of the CT scan and the slice location in the CT scan into the deep features (FC layer) as shown in Figure 4. The aim of the second task of the multi-tasks is to make the model aware of the morphological changes of the slice order in the CT scan. Both tasks use the focal loss function [35] with gamma equal to 1.5. The overall loss is the sum of the two tasks’ loss with weights of 1 and 0.3 for the first and secondary task, respectively, thus giving more importance to the main classification task (classifying the slice into normal, COVID-19, or Cap).

Since the augmented data are used in this scenario, we fine-tuned the CNN models for only 10 epochs with the Adam optimizer [36]. The initial learning rate is 0.0001, which decays by 0.1 after 3 epochs, followed by another decay of 0.1 after 6 epochs. The obtained results are summarized in Table 4. From these results, we notice that the architectures achieved a close result on classifying the slices with slightly better performance by ResneXt-50 architecture. Compared with the results of the previous scenario (Table 3), we notice that the performance improved for all CNN architectures.

**Figure 9:**
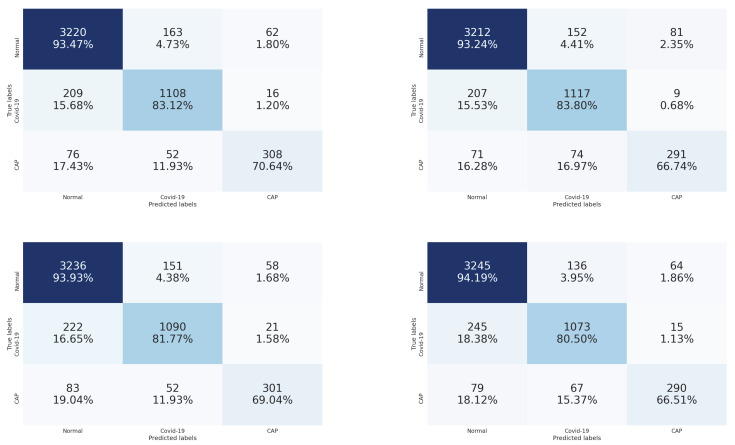
Confusion matrices of the three classes of COVID-19 testing data using ResneXt-50, Densenet-161, Inception-v3, and Wide-Resnet-50, respectively. The vertical axis is for the true classes, and the horizontal axis is for the predicted classes.

Figure 9 contains the confusion matrices of the trained CNN architectures (ResneXt-50, Densenet-161, Inception-v3, and Wide-Resnet-50) on the validation data. From these confusion matrices, we notice that Wide-Resnet-50, Densenet-161, and ResneXt-50 architectures achieved the best performance on the recognition of normal (94.19%), COVID-19 (83.80%), and Cap slices (70.64%), respectively. In this scenario, we observe that the best recognition rate of the Cap slices has improved. Subsequently, the multi-tasks strategy is efficient for the hardest class recognition.

#### 4.2.5. Patient-Level Classification

In this section, we used the trained CNN architectures (ResneXt-50, Densenet-161, Inception-v3, and Wide-Resnet-50) using the multi-tasks strategy for slice-level classification. Then, we used the prediction percentage of the three classes within the CT scan regions (each CT scan is divided into 20 regions) to train and test two classifiers, which are SVM and XG-boost, for CT scan classification. The obtained results on the validation data are summarized in Table 5. From these results, we notice that the Inception-v3 features with the XG-boost classifier achieved the best performance compared with the other architectures’ features. On the other hand, combining all four models’ features with the XG-boost classifier achieved the best performance. In contrast, combining features with the SVM classifier achieved the same result as the Wide-Resnet-50 features. This proves the efficiency of using the XG-boost classifier for single architecture features as well as combining all architectures’ features.

**Figure 10:**
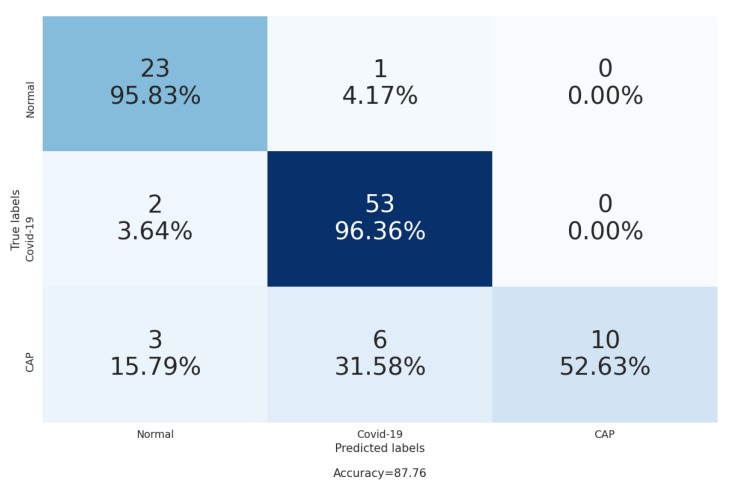
The confusion matrix of the CT scans classification on the validation data [6].

From the confusion matrix in Figure 10, we notice that our approach achieved high accuracy in the recognition of normal and COVID-19 CT scans, where it achieved 95.83% and 96.36%, respectively. On the other hand, our approach achieved 52.63% for the recognition of Cap CT scans, and this because the Cap class has less training data for both slice-level and patient-level stages than the normal and COVID-19 classes.

### 4.3. Testing Data

**Figure 11:**
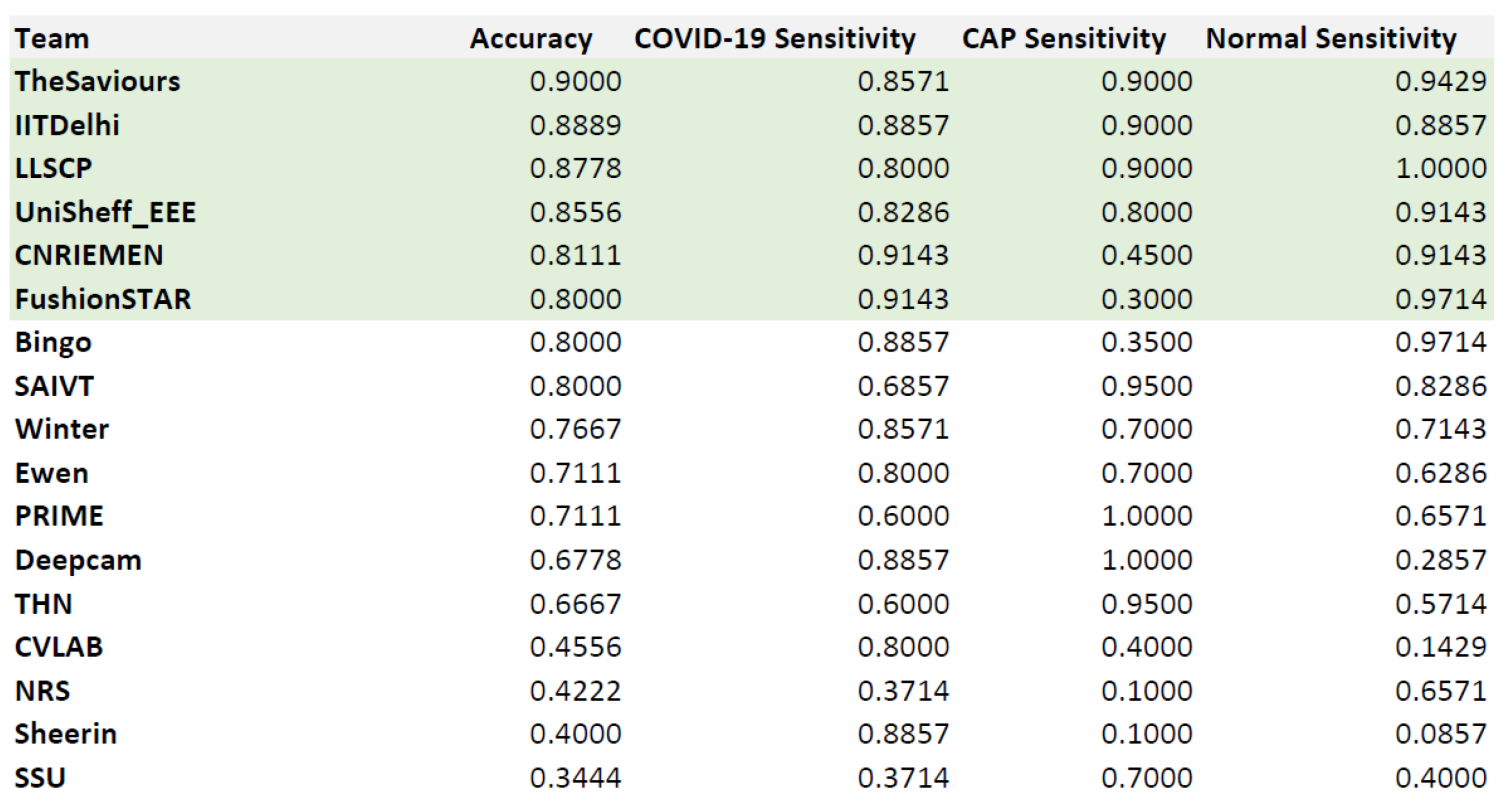
The final results of the three test sets.

In the SPGC on COVID-19 challenge, there are 17 participant teams. The testing data labels are not known by all participating teams. The overall results and rankings of the 17 teams are illustrated in Figure 11. From these results, our approach achieved fifth place in the SPGC on COVID-19 challenge. In more detail, our approach achieved 81.11% as overall accuracy and 91.43%, 45.0%, and 91.43% sensitivities for COVID-19, Cap, and normal, respectively. Compared with other participants, our approach achieved the best sensitivity for the COVID-19 class.

The results of the first, second, and third testing splits are summarized in Table 6, Table 7 and Table 8, respectively. From these tables, we notice that our approach achieved second place in the first and second testing splits. On the other hand, our approach achieved 10th place in the third testing split. Despite our rank in this testing split, our approach recognized the COVID-19 and normal cases well. In this testing split, most of the CT scans have a history of heart disease or operation with an abnormal manifestation related to a non-infection disease. This highly influenced our recognition approach for CAP cases.

### 4.4. Segmentation Influence

**Figure 12:**
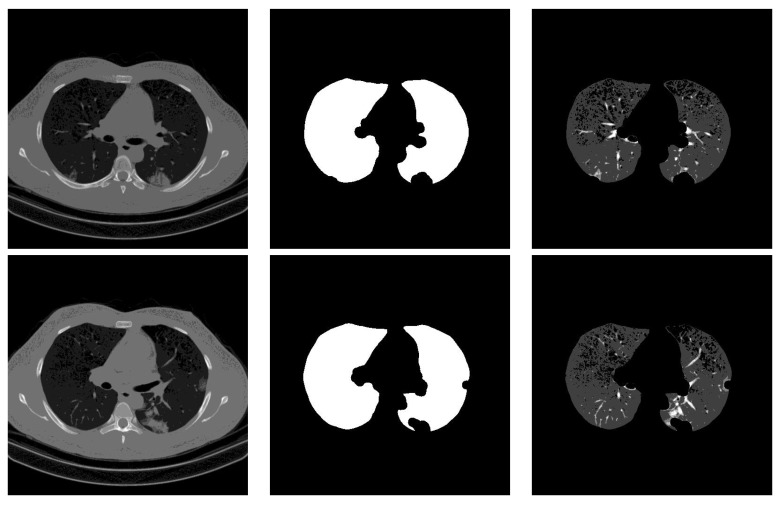

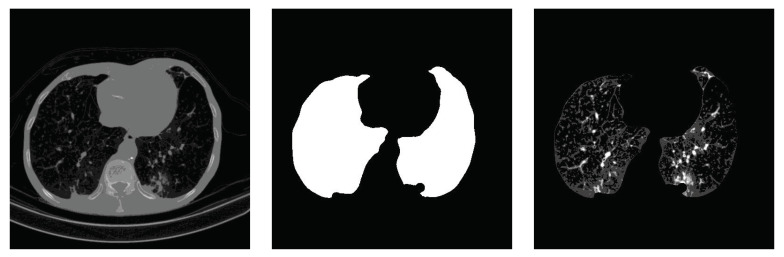
Segmented lung lobes of infected slices with COVID-19 and Cap. Rows 1, 2, and 3 are for COVID-19, Cap, and Cap slices, respectively.

Stacking segmented lung lobes with the grayscale image proved its efficiency (by comparing the results of Table 1 and Table 2). Despite this considerable improvement, the recognition of Cap slices needs more improvement. Figure 12 shows some segmented slices for COVID-19 and Cap cases. From Figure 12, we notice that the segmented results considered infection parts as lung lobes especially for Cap slices (row 2 and 3 Figure 12). One possible way to improve the performance of our approach is to use a CNN-based approach that were trained on infected slices to segment the lung lobes.

### 4.5. Heat Map

**Figure 13:**
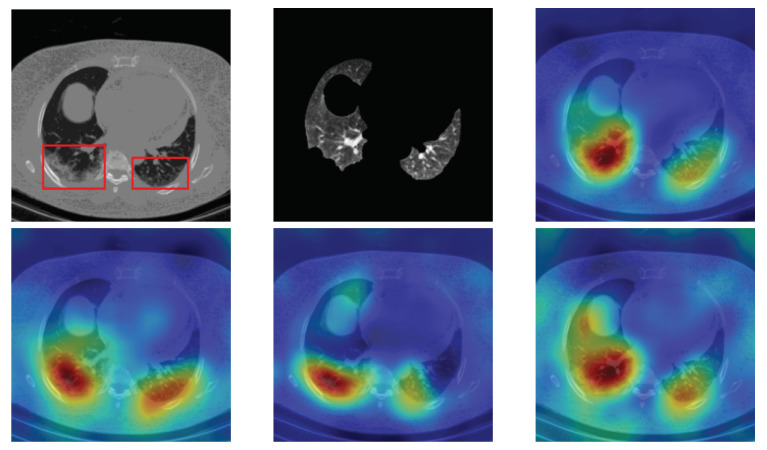

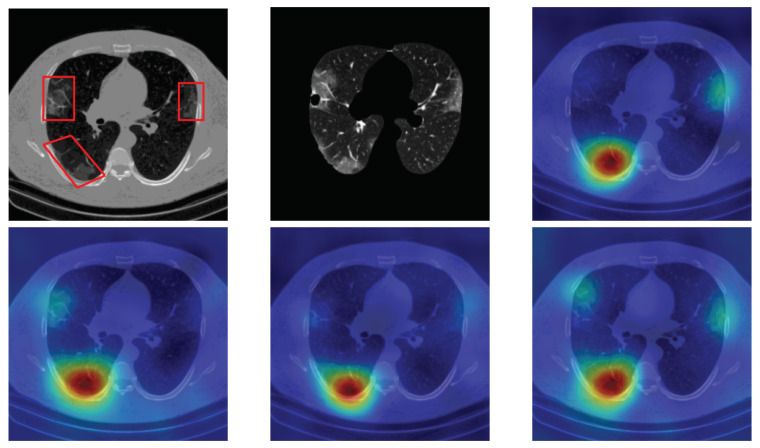
RISE heat map examples of COVID-19 slice images using the trained multi-tasks CNN architectures (ResneXt-50, Densenet-161, Inception-v3, and Wide-Resnet-50). The first example is shown in the first two rows, where the images represent the input slice image and segmented lung lobes results, followed by the heat maps of ResneXt-50, Densenet-161, Inception-v3, and Wide-Resnet-50, respectively. The second example is in rows 3 and 4.

**Figure 14:**
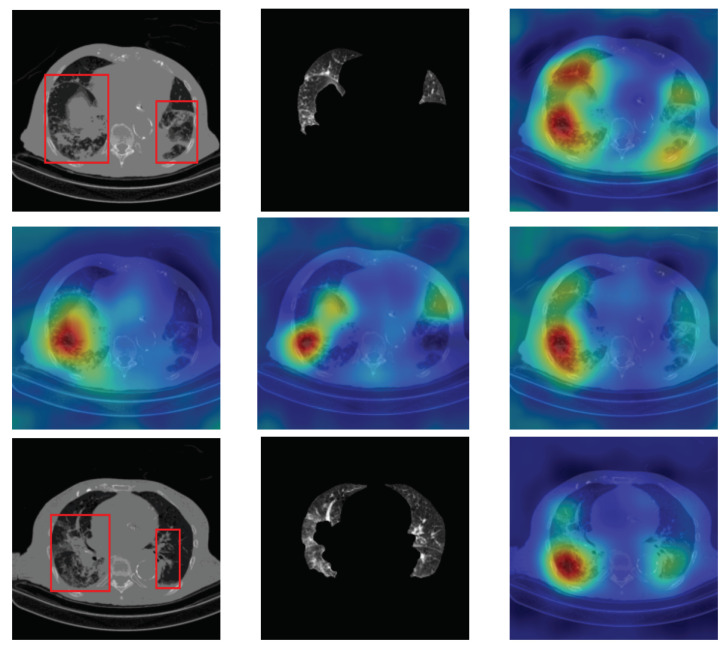

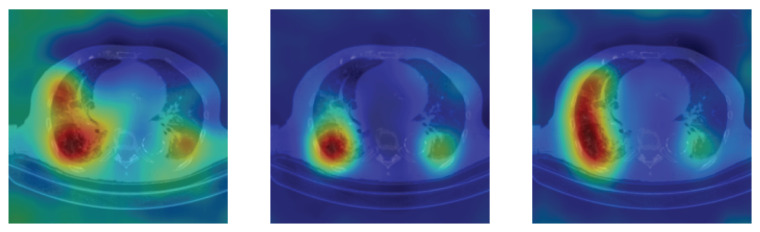
RISE heat map examples of Cap slice image using the trained multi-tasks CNN architectures (ResneXt-50, Densenet-161, Inception-v3, and Wide-Resnet-50). The first example is shown in the first two rows, where the images represent the input slice image and segmented lung lobes results, followed by the heat maps of ResneXt-50, Densenet-161, Inception-v3, and Wide-Resnet-50, respectively. The second example is in rows 3 and 4.

**Figure 15:**
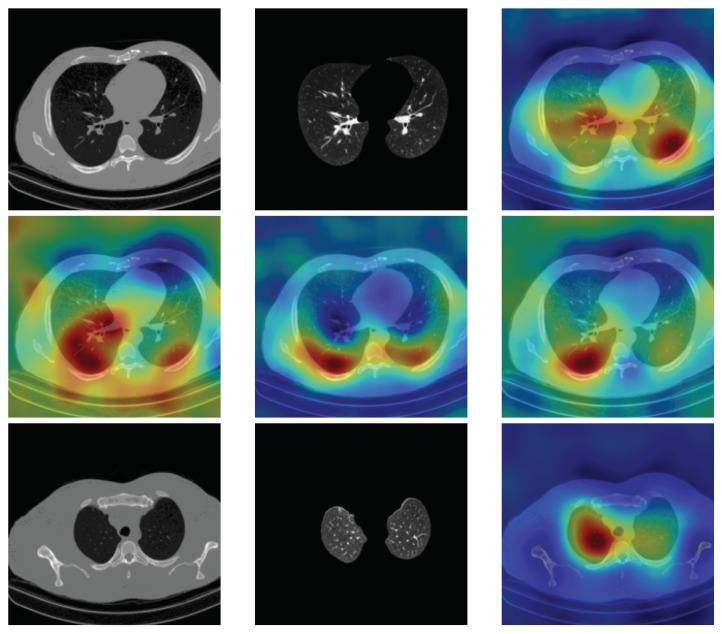

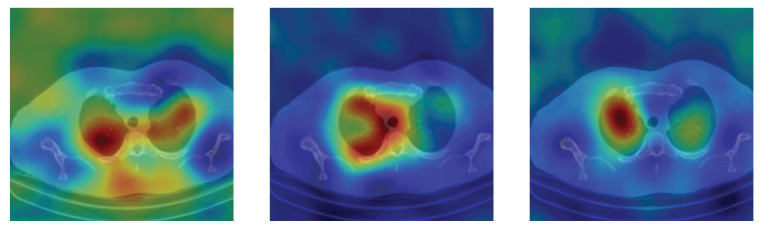
RISE heat map examples of normal slice image using the trained multi-tasks CNN architectures (ResneXt-50, Densenet-161, Inception-v3, and Wide-Resnet-50). The first example is shown in the first two rows, where the images represent the input slice image and segmented lung lobes results, followed by the heat maps of ResneXt-50, Densenet-161, Inception-v3, and Wide-Resnet-50, respectively. The second example is in rows 3 and 4.

To have more intuition about the most important lung regions that the CNN architectures consider to classify the slice images, we used the randomized input sampling for explanations (RISE) approach [37]. Figure 13, Figure 14 and Figure 15 consist of two heat map examples of COVID-19, Cap, and normal cases, respectively. In the first example from Figure 13, we notice that despite the lung segmentation being not fully correct (it misses a considerable part of the infected region), the four CNN architectures gave more importance to the infected regions and to the lung regions in general. In the second example from Figure 13, we observe that the lung lobes were precisely segmented, and the heat maps of the four CNN architectures were well defined the infected regions, especially the Wide-Resnet-50 architecture. From both Cap examples in Figure 14, we notice that despite the lung segmentation missing considerable infected parts as well as the lung lobe regions, the heat maps of all CNN architectures give more importance to the infected region and to the lung lobes in general. This proves that the trained CNN architecture is able to define the regions of interest even when the lung lobes segmentation is not good. For the normal slice examples in Figure 15, the trained CNN architectures gave more importance to the lung lobes, especially the lower region, since the infection usually occurs there. The heat maps of COVID-19, Cap, and normal cases prove that the CNN architectures learned precisely where to look to identify the infection from the slice images.

## 5. Conclusions

In this paper, we presented our proposed approach (CNR-IEMN) for the 2021 COVID-19 SPGC challenge, which aims to classify volumetric CT scans into normal, COVID-19, or Cap classes. To deal with the variation of the CT scans’ number of slices, we proposed a two-stage deep-learning-based approach: slice-level and patient-Level classification. In the slice-level classification stage, the slice images are classified into one of three classes (normal, COVID-19, or Cap). To this end, we trained four CNN architectures (ResneXt-50, Densenet-161, Inception-v3, and Wide-Resnet-50) using multi-tasks and data augmentation strategies. As input to these CNN architectures, we stacked the grayscale slice image with its lung lobe segmentation. In the second stage, we used the trained CNN architectures to extract features for the whole CT scan to train the XG-boost model. The extracted features are the percentages of the predicted classes within the CT scan regions.

To evaluate the performance of our approach, we used 2021 COVID-19 SPGC challenge validation data for both slice-level and patient-level evaluations. In addition, the three test sets of the 2021 COVID-19 SPGC challenge were used for patient-level evaluation. Our approach achieved 88.91% and 87.75% as overall accuracies on the validation data for slice-level and patient-level classification, respectively. The heat maps showed that the four trained CNN models precisely located the infection in both COVID-19 and Cap slices. On the other hand, our approach achieved fifth place on the three test datasets of SPGC in the COVID-19 challenge, where our approach achieved the best result for COVID-19 sensitivity. In more detail, the overall accuracy is 81.11% with sensitivities of 91.43%, 45%, and 91.43% for normal, COVID-19, and Cap, respectively. In addition, our approach achieved second place on two of the three testing sets.

To improve the results, especially for the Cap class, we suggest using more Cap CT scans for the slice-level and patient-level classification. Another solution to improve the performance of our approach is to use a CNN-based approach trained on infected slices to segment the lung lobes.

## Figures and Tables

**Table 1:** Validation slice-level classification results using stacked grayscale images with four backbone CNN architectures (ResneXt-50, Densenet-161, Inception-v3, and Wide-Resnet-50).

Model	Slice-Level Classification (%)
ResneXt-50	80.80
Densenet-161	81.81
Inception-v3	81.78
Wide-Resnet-50	80.72

**Table 2:** Validation slice-level classification results using stacked grayscale image with four backbone CNN architectures (ResneXt-50, Densenet-161, Inception-v3, and Wide-Resnet-50).

Model	Slice-Level Classification (%)
ResneXt-50	87.86
Densenet-161	88.10
Inception-v3	88.03
Wide-Resnet-50	87.76

**Table 3:** Validation slice-level classification results using augmented stacked gray and segmented images scenario with four backbone CNN architectures (ResneXt-50, Densenet-161, Inception-v3, and Wide-Resnet-50).

Model	Slice-Level Classification (%)
ResneXt-50	88.51
Densenet-161	88.42
Inception-v3	88.64
Wide-Resnet-50	87.97

**Table 4:** Validation of slice-level classification results using multi-tasks learning with four backbone CNN architectures (ResneXt-50, Densenet-161, Inception-v3, and Wide-Resnet-50) [6].

Model	Slice-Level Classification (%)
ResneXt-50	88.91
Densenet-161	88.61
Inception-v3	88.74
Wide-Resnet-50	88.37

**Table 5:** Validation CT scan classification results using SVM and XG-boost with different grouping percentages’ features (ResneXt-50, Densenet-161, Inception-v3, and Wide-Resnet-50 grouping percentages features) and their combination [6].

Model	CT Scans Classification (%)	
	SVM	XG-Boost
ResneXt-50 features	81.63	79.59
Densenet-161 features	82.65	82.65
Inception-v3 features	82.65	85.71
Wide-Resnet-50 features	80.61	80.61
CNR-IEMN	80.61	87.75

**Table 6:** First testing split results.

Team	COVID-19	Cap	Normal	Total
TheSaviours	9/10	8/10	9/10	26/30
**CNR-IEMN**	8/10	7/10	9/10	24/30
IITDelhi	9/10	8/10	6/10	23/30
UniSheff_EEE	9/10	7/10	7/10	23/30
Bingo	10/10	0/10	10/10	20/30
FushionSTAR	10/10	0/10	10/10	20/30

**Table 7:** Second testing split results.

Team	COVID-19	Normal	Total
LLSCP	15/15	15/15	30/30
**CNR-IEMN**	14/15	14/15	28/30
FushionSTAR	14/15	14/15	28/30
UniSheff_EEE	13/15	15/15	28/30
SAIVT	13/15	14/15	27/30
Bingo	12/15	15/15	27/30

**Table 8:** Third testing split results.

Team	COVID-19	Cap	Normal	Total
IITDelhi	10/10	10/10	10/10	30/30
LLSCP	9/10	10/10	10/10	29/30
TheSaviours	9/10	9/10	9/10	29/30
Deepcam	10/10	10/10	8/10	28/30
Ewen	10/10	9/10	8/10	27/30
..	..	..	..	..
**CNR-IEMN**	10/10	2/10	9/10	21/30

## Data Availability

The used database were provide by the organizers of the 2021 COVID-19 SPGC challenge.

## Abbreviations

The following abbreviations are used in this manuscript:

CNN	Convolutional Neural Network
RT-PCR	Reverse Transcription Polymerase Chain Reaction
SVM	Support Vector Machine
